# Supplementary material for: Virus discovery in all three major lineages of terrestrial arthropods highlights the diversity of single-stranded DNA viruses associated with invertebrates
Source: PeerJ. 2018 Oct 11;6:e5761. doi: 10.7717/peerj.5761 (PMC6186406; doi:10.7717/peerj.5761)
Supplement: Supplemental Information 3 — Branch colors distinguish sequences associated with various types of organisms and environmental sources. Bars on the right indicate clades representing genomovirus genera and unclassified sequences. Rep sequences representing Gemygorvirus (accessions: KF371632, KT862254, KT862238, KT862239, JN704610, KT732790, KT732791, KJ413144, KJ547635), Gemyduguivirus (accessions: JX185428, KY312558, KY230613), and Gemykrogvirus (accessions: KJ547634, MF327559, KJ938717, LK931484) species were merged by clade. Reps representing members from the family Geminiviridae were used as an outgroup. Genomovirus Reps identified in this study are named and highlighted with schematics of terrestrial arthropods from which they were identified, including viruses associated with sierra dome spiders (SdSACV), pimoid spiders (PiSACV), tubeweb spiders (TuwSACV), grasshoppers (GhACV) and termites (TACV). Viruses identified in multiple species of spiders are identified as spider associated circular viruses (SACV). Branches with <70% Shimodaira–Hasegawa (SH)-like support were collapsed. Arthropod silhouettes credit: Shutterstock vector library at https://www.shutterstock.com. [file peerj-06-5761-s003.pdf]

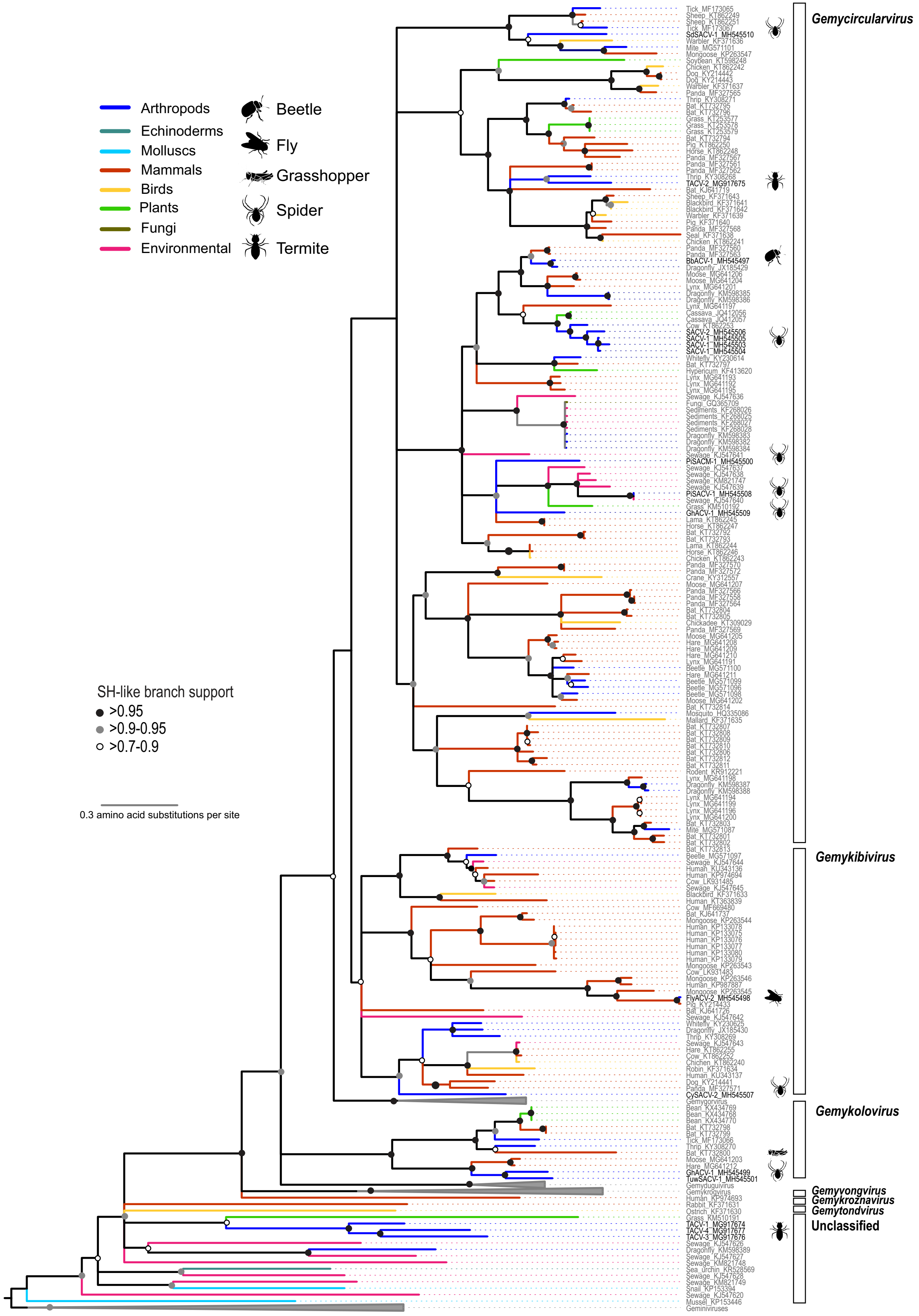
Arthropods
Echinoderms
Molluscs
Mammals
Birds
Plants
Fungi
Environmental
Beetle
Fly
Grasshopper
Spider
Termite
SH-like branch support
>0.95
>0.9-0.95
>0.7-0.9
0.3 amino acid substitutions per site
Gemycircularvirus
Gemykibivirus
Gemykolovirus
Gemyvongvirus
Gemykroznavirus
Gemytondvirus
Unclassified
Tick_MF173065
Sheep_KT862249
Sheep_KT862251
Tick_MF173067
SdSACV-1_MH545510
Warbler_KF371636
Mite_MG571101
Mongoose_KP263547
Soybean_KT598248
Chicken_KT862242
Dog_KY214442
Dog_KY214443
Warbler_KF371637
Panda_MF327565
Thrip_KY308271
Bat_KT732795
Bat_KT732796
Grass_KT253577
Grass_KT253578
Grass_KT253579
Bat_KT732794
Pig_KT862250
Horse_KT862248
Panda_MF327567
Panda_MF327561
Panda_MF327562
Thrip_KY308268
TACV-2_MG917675
Bat_KJ641719
Sheep_KF371643
Blackbird_KF371641
Blackbird_KF371642
Warbler_KF371639
Pig_KF371640
Panda_MF327568
Seal_KF371638
Chicken_KT862241
Panda_MF327560
Panda_MF327563
BbACV-1_MH545497
Dragonfly_JX185429
Moose_MG641206
Moose_MG641204
Lynx_MG641201
Dragonfly_KM598385
Dragonfly_KM598386
Lynx_MG641197
Cassava_JQ412056
Cassava_JQ412057
Cow_KT862253
SACV-2_MH545506
SACV-1_MH545505
SACV-1_MH545503
SACV-1_MH545504
Whitefly_KY230614
Bat_KT732797
Hypericum_KF413620
Lynx_MG641193
Lynx_MG641192
Lynx_MG641195
Sewage_KJ547636
Fungi_GQ365709
Sediments_KF268026
Sediments_KF268025
Sediments_KF268027
Sediments_KF268028
Dragonfly_KM598383
Dragonfly_KM598382
Dragonfly_KM598384
Sewage_KJ547641
PiSACM-1_MH545500
Sewage_KJ547637
Sewage_KJ547638
Sewage_KM821747
Sewage_KJ547639
PiSACV-1_MH545508
Sewage_KJ547640
Grass_KM510192
GhACV-1_MH545509
Lama_KT862245
Horse_KT862247
Bat_KT732792
Bat_KT732793
Lama_KT862244
Horse_KT862246
Chicken_KT862243
Panda_MF327570
Panda_MF327572
Crane_KY312557
Moose_MG641207
Panda_MF327566
Panda_MF327558
Panda_MF327564
Bat_KT732804
Bat_KT732805
Chickadee_KT309029
Panda_MF327569
Moose_MG641205
Hare_MG641208
Hare_MG641209
Hare_MG641210
Lynx_MG641191
Beetle_MG571100
Hare_MG641211
Beetle_MG571099
Beetle_MG571096
Beetle_MG571098
Moose_MG641202
Bat_KT732814
Mosquito_HQ335086
Mallard_KF371635
Bat_KT732807
Bat_KT732808
Bat_KT732809
Bat_KT732810
Bat_KT732806
Bat_KT732812
Bat_KT732811
Rodent_KR912221
Lynx_MG641198
Dragonfly_KM598387
Dragonfly_KM598388
Lynx_MG641194
Lynx_MG641199
Lynx_MG641196
Lynx_MG641200
Bat_KT732803
Mite_MG571087
Bat_KT732801
Bat_KT732802
Bat_KT732813
Beetle_MG571097
Sewage_KJ547644
Human_KU343136
Human_KP974694
Cow_LK931485
Sewage_KJ547645
Blackbird_KF371633
Human_KT363839
Cow_MF669480
Bat_KJ641737
Mongoose_KP263544
Human_KP133078
Human_KP133075
Human_KP133076
Human_KP133077
Human_KP133080
Human_KP133079
Mongoose_KP263543
Cow_LK931483
Mongoose_KP263546
Human_KP987887
Mongoose_KP263545
FlyACV-2_MH545498
Pig_KY214433
Bat_KJ641726
Sewage_KJ547642
Whitefly_KY230625
Dragonfly_JX185430
Thrip_KY308269
Sewage_KJ547643
Hare_KT862255
Cow_KT862252
Chichen_KT862240
Robin_KF371634
Human_KU343137
Dog_KY214441
Panda_MF327571
CySACV-2_MH545507
Gemygorvirus
Bean_KX434769
Bean_KX434768
Bean_KX434770
Bat_KT732798
Bat_KT732799
Tick_MF173066
Thrip_KY308270
Bat_KT732800
Moose_MG641203
Hare_MG641212
GhACV-1_MH545499
TuwSACV-1_MH545501
Gemyduguivirus
Gemykrogvirus
Human_KP974693
Rabbit_KF371631
Ostrich_KF371630
Grass_KM510191
TACV-1_MG917674
TACV-4_MG917677
TACV-3_MG917676
Sewage_KJ547626
Dragonfly_KM598389
Sewage_KJ547627
Sewage_KM821748
Sea_urchin_KR528569
Sewage_KJ547628
Sewage_KM821749
Snail_KP153394
Sewage_KJ547620
Mussel_KP153446
Geminiviruses
